# Neuro–immune–tumor axis in gliomas: a review of mechanisms, models, and translational opportunities

**DOI:** 10.3389/fimmu.2025.1682322

**Published:** 2025-10-08

**Authors:** Lu Xu, Shuangyu Chen, Yixin Fu, Tingting Zhou, Jianghao Yu, Jiayang Li, Wei Chen

**Affiliations:** ^1^ The First Affiliated Hospital of Zhejiang Chinese Medical University (Zhejiang Provincial Hospital of Chinese Medicine), Hangzhou, Zhejiang, China; ^2^ School of Medical Technology and Information Engineering, Zhejiang Chinese Medical University, Hangzhou, Zhejiang, China; ^3^ Department of Neurosurgery, The Second People’s Hospital of Yibin, Yibin, Sichuan, China

**Keywords:** neuro–immune–tumor axis, glioma, tumor microenvironment, neuronal activity, immune suppression, AMPA receptor, immunotherapy

## Abstract

Neuroimmuno-oncology is an emerging interdisciplinary field that explores the complex interactions among the nervous system, the immune system, and tumor cells within the tumor microenvironment (TME). Recent studies have underscored the critical role of neurons in gliomas, where synaptic signaling and the release of neurotrophic factors contribute not only to tumor progression but also to mechanisms of immune evasion. Neurotransmitters such as glutamate and gamma-aminobutyric acid (GABA), along with neuron-derived factors including brain-derived neurotrophic factor (BDNF) and neuroligin-3 (NLGN3), have been shown to modulate immune cell function and promote the formation of an immunosuppressive TME. In particular, neuronal electrical activity mediated through α-amino-3-hydroxy-5-methyl-4-isoxazolepropionic acid receptor (AMPAR) signaling facilitates immune escape in glioma cells, leading to the development of an “immune-excluded” phenotype that compromises the efficacy of immunotherapy. Therapeutic strategies that combine AMPAR antagonists with immune checkpoint inhibitors—alongside neuromodulatory techniques such as repetitive transcranial magnetic stimulation (rTMS) or deep brain stimulation (DBS)—hold potential to reprogram the neuro–immune–tumor axis, remodel the immune landscape, and improve immunotherapy responses in central nervous system malignancies. Advancing our understanding of how neuronal activity regulates the glioma immune microenvironment may open new avenues for precision-targeted therapeutic approaches in neuro-oncology.

## 1 Introduction

Neuroimmuno-oncology is an emerging interdisciplinary field that investigates the intricate interplay between the nervous system, the immune system, and cancer (1). As neuroscience, immunology, and oncology continue to converge, accumulating evidence suggests that the nervous system is not merely a conduit for information transmission but also an active regulator of tumor initiation, progression, and immune evasion (2–4). This field provides a novel framework to elucidate the mechanisms underlying immunologically “cold” tumors, supporting the development of combinatorial interventions aimed at enhancing immunotherapy responsiveness (5, 6). It is increasingly recognized as a promising direction for innovation in cancer treatment. The nervous system constitutes a critical component of the tumor microenvironment (TME) (7). Functionally active neurons can engage tumor cells through electrical and chemical synapses or modulate tumor behavior via the paracrine release of neuromodulatory factors (8, 9), including neuroligin-3 (NLGN3) (10), brain-derived neurotrophic factor (BDNF) (11, 12), glutamate (13), and norepinephrine (5). These signals reshape tumor cell metabolism, oncogenic signaling pathways, and immune phenotypes. Moreover, neuronal activity has been shown to promote the upregulation of immune checkpoints and the recruitment of immunosuppressive cell populations such as regulatory T cells (Tregs) and myeloid-derived suppressor cells (MDSCs), thereby fostering an immunosuppressive TME (3).

**Figure 1:**
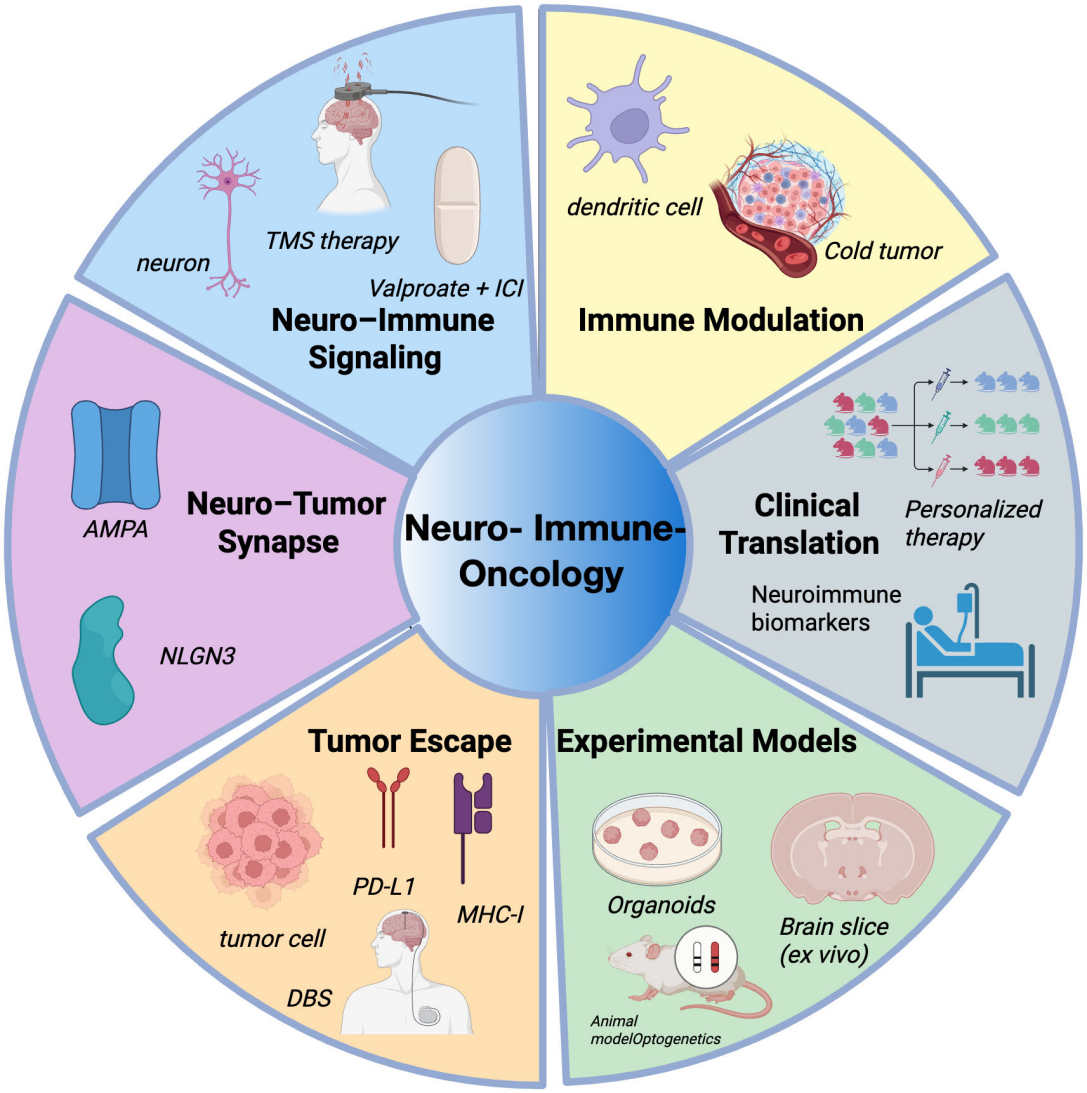
Overview of major research areas in neuro–immune–oncology.

Among central nervous system malignancies, gliomas represent a prototypical model of neuron-cancer crosstalk (10). Recent studies have demonstrated that glioma cells can form bona fide synaptic connections with active neurons, establishing α-amino-3-hydroxy-5-methyl-4-isoxazolepropionic acid receptor (AMPAR)–dependent synaptic networks (13). Neuronal activity synchronizes calcium signaling within the tumor, enhances intercellular coupling, and activates the phosphoinositide 3-kinase (PI3K) – mechanistic target of rapamycin (mTOR) axis through NLGN3, driving glioma proliferation via a feedforward loop (10, 14). These findings highlight a central role for neuronal input in glioma progression and suggest novel therapeutic targets within neuron–tumor signaling pathways. Beyond tumor-intrinsic effects, neurons also exert immunomodulatory influence. In various malignancies, neuron-derived neurotransmitters and neurotrophic factors modulate immune cell behavior through receptor-mediated signaling, orchestrating the transition between immune-inflamed (“hot”) and immune-excluded/deserted (“cold”) tumor states (15). This neuron–immune–tumor triad offers a mechanistic explanation for immune resistance in certain tumors and points toward rational strategies to combine neural modulation with immune interventions (3, 6). As research in neuroimmuno-oncology progresses, this field is poised to enhance the precision of immunotherapies, overcome resistance, and improve outcomes across multiple tumor types. In this review, we explore the molecular mechanisms through which neurons regulate anti-tumor immunity and discuss their potential implications for cancer immunotherapy (**Figure 1**).

## 2 Neuronal activity and glioma progression

The progression of high-grade gliomas is closely associated with neuronal activity. Clinical observations indicate that brain regions exhibiting higher neuronal firing rates frequently correlate with more aggressive tumor phenotypes and significantly reduced patient survival, highlighting the crucial role of neuronal excitability in glioma biology (4). Glioma cells preferentially migrate toward neuron-rich regions and actively establish synapse-like structural connections with neurons, enabling excitatory neuronal signals to directly stimulate tumor cells (16, 17). These “neuron–glioma synapses” predominantly rely on glutamate-mediated neurotransmission, activating calcium-permeable α-amino-3-hydroxy-5-methyl-4-isoxazolepropionic acid receptors (CP-AMPARs) on glioma cell membranes. This activation induces sustained membrane depolarization and downstream oncogenic signaling cascades, thereby enhancing glioma cell proliferation and invasiveness (13, 18). Furthermore, neurotransmitters such as glutamate and norepinephrine accumulate within the synaptic microenvironment, further elevating neuronal excitability. Concurrently, these neurotransmitters stimulate glioma cells through corresponding receptors, exacerbating metabolic reprogramming and malignant progression (13, 17, 19).

Intriguingly, glioma cells themselves may acquire neuron-like electrophysiological properties, actively participating within neuronal circuits and further integrating into existing neural networks. This neuronal phenotype potentially allows glioma cells to generate autonomous rhythmic activity, amplifying their invasiveness and resistance to therapy (9). Recent studies have also suggested that glioma-induced remodeling of neuronal circuits can reciprocally enhance neuronal hyperexcitability, establishing a self-reinforcing feedback loop between tumor growth and neuronal activity (4). Such a mechanism might contribute to both tumor progression and treatment resistance, underscoring the complexity and therapeutic challenge posed by neuron–glioma interactions. Collectively, these findings reveal the bidirectional and dynamic nature of neuron–glioma interactions. Insights into mechanisms such as synapse formation, neurotransmitter metabolism, and receptor-mediated signaling pathways, as well as the reciprocal modulation of neuronal circuits by gliomas, could provide novel therapeutic strategies aimed at disrupting pathological neuron–glioma crosstalk and effectively impeding tumor progression (11, 13, 14).

## 3 Mechanisms of neuromodulation of the TME

### 3.1 Effects of neurotransmitters on immune cells

Neurotransmitters, while classically mediating neural communication, also serve as critical regulators of immune responses by modulating the differentiation, activation, and function of immune cells (20). Key mediators such as glutamate, gamma-aminobutyric acid (GABA), and acetylcholine (ACh) shape the immune phenotype of T cells, macrophages, and dendritic cells through receptor-dependent signaling within the tissue microenvironment (19, 21, 22). Furthermore, neurotransmitters facilitate crosstalk and positive feedback loops between immune cells and the sympathetic or parasympathetic nervous systems, enabling dynamic regulation of immune and inflammatory states (5, 23).

In addition to glutamate, several other neurotransmitter systems have been implicated in shaping immune cell behavior. GABA, the primary inhibitory neurotransmitter in the CNS, can suppress T cell proliferation and cytokine production via GABA_A and GABA_B receptor signaling on lymphocytes and myeloid cells (24, 25). GABAergic signaling has been associated with increased Treg differentiation and impaired cytotoxic function, potentially contributing to immune escape in the tumor microenvironment (26, 27). Cholinergic signaling, mediated by ACh, modulates macrophage activation states and promotes anti-inflammatory responses through α7 nicotinic acetylcholine receptors (α7 nAChRs). Cholinergic macrophages have been shown to regulate peritoneal inflammation and may play roles in tumor immunity. Norepinephrine, a key neurotransmitter of the sympathetic nervous system, exhibits context-dependent effects: chronic adrenergic signaling may suppress CD8+ T cell activity and enhance immunosuppressive cell recruitment (5), whereas acute stimulation may facilitate dendritic cell mobilization and antigen presentation. These findings underscore the complexity and diversity of neurotransmitter-mediated immune regulation beyond glutamatergic pathways.

### 3.2 Neuronal regulation of the immunosuppressive microenvironment

The tumor immune microenvironment (TIME) plays a central role in shaping antitumor immunity, influencing therapeutic responses, disease progression, and clinical outcomes (28). Far beyond a simple aggregation of malignant cells, the TME comprises a complex network of infiltrating or resident immune cells—including T cells, MDSCs, Tregs, and tumor-associated macrophages (TAMs)—alongside stromal components such as cancer-associated fibroblasts (CAFs), astrocytes, and neurons (29). Accumulating evidence indicates that neurons actively contribute to the remodeling of the TME (3). Functionally active neurons not only engage in direct communication with tumor cells via electrical and chemical synapses but also influence local immune dynamics through the paracrine secretion of neuron-derived immunomodulatory molecules (30, 31). Among these, NLGN3 and BDNF have emerged as key neuronal mediators that drive both tumor progression and immunosuppressive remodeling (11, 32). In high-grade gliomas such as glioblastoma (GBM), neuronal activity promotes tumor growth through the secretion of soluble NLGN3, which activates the PI3K–mTOR signaling cascade and triggers feedforward expression of NLGN3 within glioma cells, thereby amplifying their proliferative potential (10, 14). Beyond promoting tumor proliferation, NLGN3 and BDNF have also been implicated in the reprogramming of the tumor immune microenvironment, potentially facilitating the recruitment and activation of immunosuppressive populations such as Tregs and MDSCs (3, 12). This neuron–immune axis forms a positive feedback loop that promotes immune evasion and sustains tumor growth (3, 33, 34).

Moreover, neuronal activity has been increasingly recognized as playing a pivotal role in the establishment of the “immune-cold” tumor phenotype (7). Compared to immune-inflamed tumors, immune-cold tumors are typically characterized by reduced immune infiltration, impaired antigen presentation, and T cell exhaustion (28, 35). Further investigations have demonstrated that heightened neuronal activity can upregulate the expression of immune-regulatory factors such as C–C motif chemokine ligand 2(CCL2), interleukin-1 beta (IL-1β), and prostaglandin E2 (PGE2), thereby promoting the accumulation of MDSCs, Tregs, and M2-polarized macrophages within the TME (5, 23). This process not only exacerbates immunosuppression but also impairs T cell activation, dampening the antitumor immune response. Moreover, single-cell transcriptomic analyses have revealed a strong association between neurogenic signaling pathways and immune exclusion signatures in immune-cold tumors, suggesting that the nervous system actively contributes to immune evasion through spatial and functional modulation of the TME (3, 36).

### 3.3 Immune escape mechanisms in glioma cells after neural influence

In high-grade gliomas, hyperactive neurons establish functional synaptic connections with tumor cells, delivering excitatory postsynaptic currents (EPSCs) mediated by CP-AMPARs (8, 13, 18). These EPSCs induce sustained membrane depolarization, thereby promoting tumor cell proliferation and invasiveness (10, 11, 18). Concurrently, neuronal secretion of BDNF activates the tropomyosin receptor kinase B (TrkB) and signals through calcium/calmodulin-dependent protein kinase II (CaMKII), promoting AMPAR trafficking to the glioma cell membrane and enhancing glutamate-evoked current amplitudes (11, 13). These processes induce synaptic strengthening in glioma cells, mimicking mechanisms of physiological synaptic plasticity in the healthy brain, and contribute to malignant progression. This neuron-driven “electro-metabolic axis” not only fuels glioma progression but also impairs immune surveillance by downregulating the expression of antigen-processing components such as transporter associated with antigen processing 1 (TAP1) and transporter associated with antigen processing 2 (TAP2) through AMPAR–mediated signaling (11), and by enhancing programmed death-ligand 1 (PD-L1) expression via mTOR-dependent pathways (37). These alterations collectively diminish antigen presentation and enhance PD-1 binding affinity, contributing to an immune-evasive tumor phenotype.

Emerging evidence further highlights that gliomas hijack additional signaling axes to consolidate immune evasion. Among these, the adenosine pathway—driven by ectonucleoside triphosphate diphosphohydrolase-1 (CD39)/ecto-5’-nucleotidase (CD73) ectoenzymes and signaling through adenosine A2A receptor (A2AR)—constitutes a dominant immunosuppressive mechanism that induces T cell exhaustion and promotes regulatory cell recruitment (38, 39). Recent findings also reveal that kynurenine–aryl hydrocarbon receptor (AHR) signaling in tumor-associated macrophages upregulates CD39, thereby reinforcing adenosine production and amplifying immunosuppression within the glioma microenvironment. Collectively, these interconnected pathways converge to establish a multilayered barrier against effective antitumor immunity. These immunosuppressive mechanisms lay the groundwork for further immune exclusion orchestrated by neuronal cues.

Importantly, such electrically driven gliomas are not only highly proliferative but also exhibit a distinctly immune-excluded tumor microenvironment (11). Neuronal activity reshapes the tumor microenvironment by promoting the recruitment of immunosuppressive cell populations, including Tregs and MDSCs, and by upregulating immunomodulatory molecules such as CD73 and PD-L1, thereby suppressing effector T cell infiltration and function (5, 40, 41). Unlike peripheral tumors such as breast cancer, which rarely receive direct synaptic input, gliomas uniquely convert neuronal hyperactivity into both oncogenic and immunosuppressive signals (4, 42). This neuron-driven immunological remodeling contributes to the establishment of an exclusionary immune niche, fostering immune escape and potentially compromising the efficacy of immune checkpoint blockade therapies (37, 43). These neuromodulatory effects on the immune environment pave the way for understanding direct neuronal influence on immune escape in gliomas.

## 4 A systematic model of neural-immune-tumor interactions

In recent years, accumulating evidence has demonstrated that the nervous system contributes to tumor initiation and progression not only by modulating tumor cell behavior but also by profoundly influencing the composition and functionality of the immune system (20, 44). This has led to the conceptualization of a multidimensional regulatory framework known as the neuron–immune–tumor axis (30). Neurons, through synaptic activity, neurotrophic factors (such as BDNF, neuroligin-3), and neurotransmitters (such as norepinephrine, glutamate), can directly enhance tumor cell proliferation and invasiveness, while also indirectly promoting immune evasion by recruiting immunosuppressive cells such as Tregs and MDSCs or depleting effector T cells (5, 45, 46). Further studies have shown that neuronal signals, including BDNF and norepinephrine, can modulate the phenotype and function of microglia or dendritic cells (12, 23). For example, BDNF signaling through the TrkB receptor has been reported to influence immune-related gene expression (47). Similarly, sympathetic neuron-derived norepinephrine has been shown to upregulate the secretion of chemokines such as CCL2 within the tumor microenvironment, thereby promoting the recruitment of immunosuppressive populations including Tregs and MDSCs (5, 33). Immune cells modulated by neural inputs can, in turn, promote tumor progression through diverse mechanisms, thus forming a functional coupling loop among neurons, immune cells, and tumor cells. For example, Tregs and MDSCs secrete immunosuppressive cytokines such as interleukin-10(IL-10) and transforming growth factor beta (TGF-β) to suppress effector T cell function (34), while activated microglia release tumor-promoting factors including epidermal growth factor (EGF) and matrix metalloproteinases (MMPs), enhancing glioma cell proliferation and invasiveness (48). Additionally, tumor cells under neural influence often upregulate immune checkpoint molecules such as PD-L1 and CD73, further impairing T cell–mediated immune clearance and reinforcing immune escape (41, 49).

While neurons actively regulate immune dynamics in the tumor microenvironment, immune cells can also reciprocally influence neural activity, forming a bidirectional signaling loop. For example, microglia and peripheral immune cells release cytokines such as IL-1β, tumor necrosis factor alpha (TNF-α), and Interferon-gamma (IFN-γ) that modulate synaptic plasticity, neuronal excitability, and neurotransmitter release. Chronic neuroinflammation may result in maladaptive remodeling of neural circuits, with implications for tumor-associated seizures and cognitive dysfunction (50). Tregs and MDSCs can influence neuronal and glial signaling via immunosuppressive mediators like IL-10 and TGF-β. In addition, activated microglia and macrophages may enhance neuronal excitability or alter synaptic pruning through the release of BDNF, reactive oxygen species (ROS), or complement proteins, further reinforcing the immunosuppressive microenvironment and potentially driving neuroplastic adaptations within the tumor niche (51–53).

The increasingly delineated “neuron → immune cell → tumor cell” signaling cascade forms a core mechanism underlying the neurogenic immunosuppressive phenotype observed in gliomas. In this multicellular signaling circuit, neurons first activate immune cells via synaptic activity and soluble factors, leading to the expression of immune checkpoints or secretion of immunosuppressive molecules, which in turn facilitate tumor progression and immune evasion (36, 54). Simultaneously, tumor-associated immune cells may feedback to regulate neural activity or sustain inflammatory signaling, forming a positive feedback loop that amplifies the immunosuppressive milieu (29, 55, 56). This neuron–immune–tumor axis not only enhances our understanding of immune escape mechanisms in central nervous system tumors such as high-grade gliomas but also provides a theoretical basis and strategic direction for developing combined neuro-modulatory and immunotherapeutic approaches.

**Figure 2:**
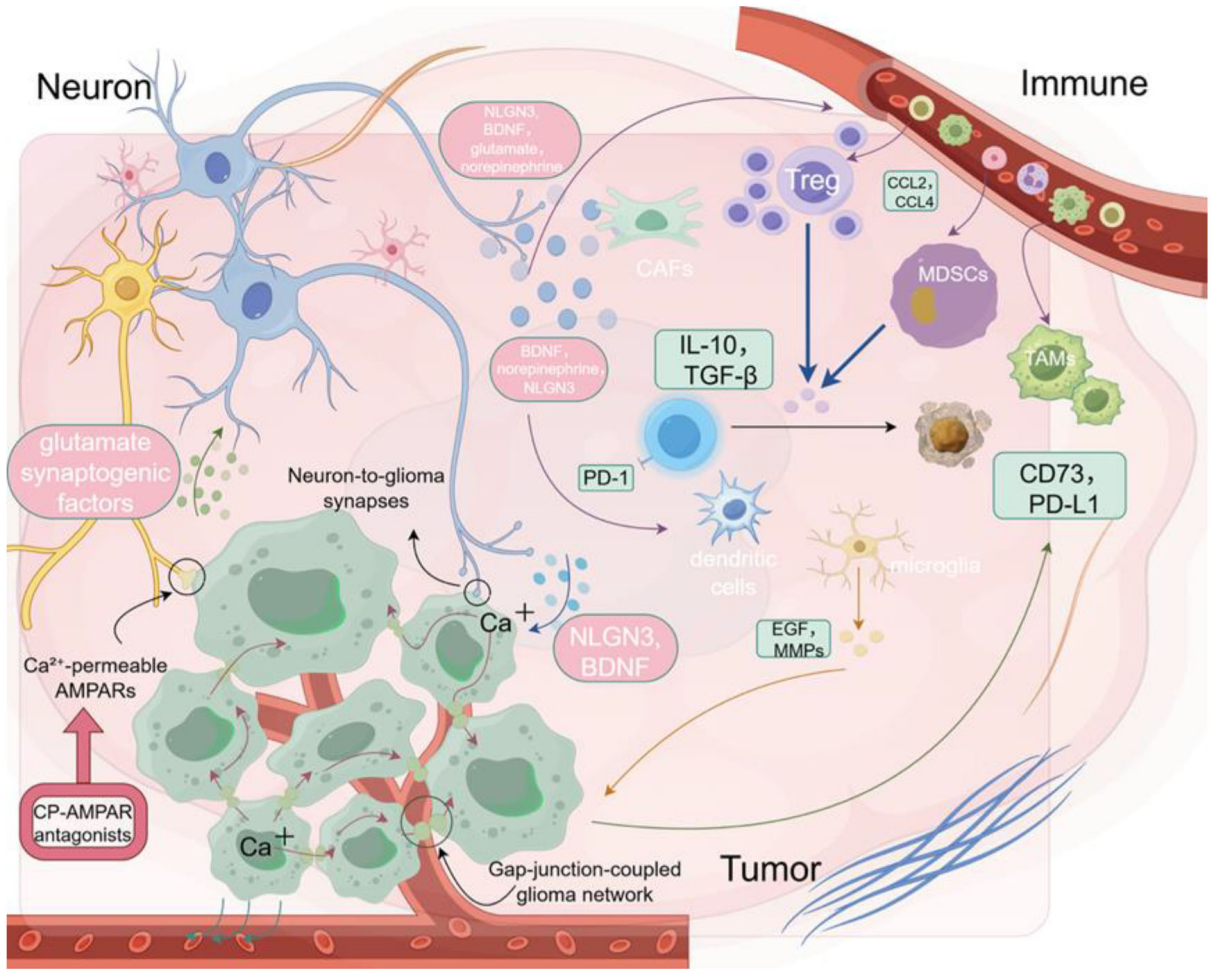
Neuron–tumor–immune interactions in the glioma microenvironment. Neurotransmitters such as glutamate and norepinephrine promote tumor growth and immune exclusion by modulating immune cell behavior.

To better investigate the dynamic interactions within the neuron–immune–tumor triad, organoids and brain organoid models are emerging as ideal experimental platforms. Compared to traditional 2D cultures or animal models, 3D organoid systems better preserve the structural architecture, gene expression profiles, and immune microenvironmental heterogeneity of primary tumors, allowing for more accurate modeling of complex interactions among tumor cells, stromal cells, and immune components (57, 58). Brain organoids further offer visualization and functional assessment of neuronal development, synaptic connectivity, and neurotransmitter release, thereby supporting the study of how neurons regulate immune cell behavior within a spatial and electrophysiological context (59–61). These models are also highly compatible with CRISPR-based gene editing, high-throughput drug screening, and single-cell omics, enabling the integration of mechanistic investigation with therapeutic target validation and holding promise for the development of individualized combination interventions (62–65). Recent technological advances have further enhanced the physiological relevance of organoid systems. For instance, 3D bioprinting allows spatially controlled deposition of induced pluripotent stem cells (iPSC)-derived neurons and immune cells using bioinks laden with functional sensory neuron populations, enabling the localized reconstruction of electrophysiologically active neural circuits and long-range axon guidance (66). The integration of microfluidic platforms permits dynamic perfusion and localized delivery of soluble factors, thereby supporting real-time tracking of neural–immune–tumor interactions under defined chemical and flow conditions (66, 67). These approaches facilitate modular and personalized modeling of synapse-driven immune modulation and offer scalable platforms for evaluating neuro-immune-targeted therapeutic strategies. Nonetheless, current organoid and brain organoid platforms still face limitations. Certain immune cell subpopulations may be lost during long-term culture, and the absence of vascular networks, neural innervation, and mechanical stress limits the faithful recapitulation of dynamic feedback processes within the neuron–immune–tumor axis (68–70). Future efforts integrating microfluidic chips, spatial transcriptomics, and multimodal imaging may enhance the physiological relevance and real-time monitoring capabilities of these models, thereby providing more accurate *in vitro* platforms for decoding neuro-immune regulation and optimizing therapeutic strategies (58) (**Figure 2**).

## 5 Clinical translation of neuro-immune therapeutics

### 5.1 Interventional neuromodulation to enhance immunotherapy

In recent years, immune checkpoint inhibitors (ICIs) have achieved significant breakthroughs in the treatment of various solid tumors (71). However, their efficacy remains limited in neurogenic “cold tumors,” such as gliomas and small-cell lung cancer, largely due to the presence of a neuronally regulated immunosuppressive TME (7). Emerging evidence indicates that neurons release neurotransmitters such as glutamate and norepinephrine, which activate AMPAR—particularly CP-AMPARs—on tumor and glial cells (13). This process promotes the recruitment of immunosuppressive cells, including Tregs and MDSCs, induces T cell exhaustion, and shapes a profoundly immune-excluding TME (18). This neuro–tumor–immune axis not only undermines T cell-mediated immunity but also facilitates immune evasion, thereby limiting the therapeutic potential of ICIs (37, 72). Targeting this axis to reprogram the immune landscape has thus emerged as a promising strategy to enhance ICI efficacy. CP-AMPAR antagonists, such as 1-naphthylacetyl spermine trihydrochloride (NASPM), selectively inhibit glutamate-induced calcium influx and neuronal excitatory signaling, effectively disrupting tumor–neuron synaptic coupling and alleviating immunosuppression at its source (40). By mitigating T cell exhaustion and restoring antigen presentation capacity, this “de-excitation” strategy establishes a more favorable immune contexture for ICIs to exert their effects—enabling a synergistic transition from neural disinhibition to immune reactivation (40). Additionally, AMPAR blockade may modulate microglial and astrocyte-mediated synaptic remodeling and immunoregulation (40, 73), potentially improving the immunological tone of the tumor microenvironment and enhancing responsiveness to immune checkpoint inhibitors.

This combinatorial strategy holds substantial clinical potential, particularly in the context of tumors characterized by heightened neural activity and immune evasion (11). Inhibiting CP-AMPARs may function as a pre-conditioning approach to disrupt neuro–tumor crosstalk, alleviate immunosuppression, and enhance tumor immunogenicity—thereby sensitizing tumors to ICIs (13, 40, 44, 73). In this reprogrammed immune landscape, ICIs may subsequently amplify antitumor responses by promoting effector T cell expansion, fostering memory formation, and supporting durable immunity (74, 75). This synergy may be especially beneficial in malignancies with elevated PD-L1 expression yet suboptimal ICI responsiveness, or in those complicated by neuroinflammatory comorbidities (54, 76). To fully realize this potential, future studies should investigate the integration of this dual-targeted approach with radiotherapy, oncolytic virotherapy, or personalized cancer vaccines, while optimizing delivery systems and therapeutic sequencing to facilitate clinical translation.

In parallel with pharmacological blockade, non-invasive neuromodulation technologies are gaining traction as complementary strategies to modulate the immune microenvironment. Repetitive transcranial magnetic stimulation (rTMS), widely used in neuropsychiatric disorders, has been shown—particularly in its low-frequency (<1 Hz) form—to suppress oncogenic signaling cascades (such as PI3K/protein kinase B (AKT) and extracellular signal-regulated kinase (ERK)/c-Jun N-terminal kinase (JNK)) and induce tumor cell apoptosis (77); its potential immunomodulatory effects, including on local T cell infiltration, remain to be further elucidated. Moreover, rTMS may influence sympathetic nervous system activity, potentially affecting norepinephrine-mediated modulation of the tumor microenvironment and thereby impacting the efficacy of immune checkpoint inhibitors (5, 78). Deep brain stimulation (DBS), as a precise and adjustable neurostimulation technique, also demonstrates potential for immune modulation by altering central excitability and inflammatory feedback (79, 80). In preclinical epilepsy models, anterior nucleus (AN)-targeted DBS significantly reduces hippocampal interleukin-6 (IL-6) levels and caspase-3 activity, indicating its anti-inflammatory and anti-apoptotic effects on the brain’s immune milieu (80). Clinical studies in Parkinson’s disease suggest that subthalamic nucleus (STN)-DBS can reduce dopaminergic medication burden, stabilize limbic system dynamics, and alleviate impulsivity-related behavioral disturbances (81). These effects may also contribute to the mitigation of neuroinflammation and psychological stress, both of which are common in cancer patients with central nervous system involvement (82). Collectively, both pharmacological AMPAR blockade and neuromodulatory interventions such as rTMS and DBS offer synergistic potential with ICIs by reconfiguring the neuro–immune axis, reversing immune suppression, and enhancing antitumor immunity. CP-AMPAR inhibition serves as a “de-excitation” strategy to suppress glutamatergic immune dampening, while electrical neuromodulation enables structural reprogramming of immune thresholds and inflammatory states (77, 79). Moving forward, the convergence of these approaches underscores the need to develop multimodal therapeutic models that integrate neural-targeted agents, neuromodulation, and immune checkpoint blockade—guided by biomarker-driven stratification, optimal treatment timing, and translational validation within the framework of precision oncology.

Beyond small molecules and electrical stimulation, biologically engineered vectors are emerging as the third pillar of neuroimmune modulation. A representative example is the oncolytic adenovirus with triple deletions expressing non-secreting interleukin-12 (Ad-TD-nsIL12), which is currently under investigation in two single-center phase I clinical trials (NCT05717712 for newly diagnosed and NCT05717699 for progressive isocitrate dehydrogenase (IDH)-wildtype gliomas) in pediatric patients. This agent not only promotes direct tumor cell lysis but also enhances local immune activation through the sustained expression of non-secreting interleukin-12 (IL-12) (83). It demonstrates the potential to reshape the immune microenvironment by increasing cytotoxic T lymphocyte infiltration and reducing immunosuppressive cell populations—effects that are particularly valuable in pediatric gliomas characterized by neural regulation and immune exclusion. Similarly, the novel agent DMAMCL, derived from the natural compound micheliolide, has shown potent antitumor activity in preclinical models by inducing apoptosis and impairing mitochondrial function in tumor cells, while simultaneously modulating nuclear factor kappa-light-chain-enhancer of activated B cells (NF-κB)–mediated inflammatory signaling (84). These emerging technologies offer scalable, biologically integrated platforms to simulate and intervene in neuroimmune dynamics, and may serve as adjuncts to ICIs by transforming cold tumor microenvironments into immunologically responsive states (**Table 1**).

**Table 1:** Interventional neuromodulation strategies enhancing immune checkpoint inhibitor (ICI) efficacy in cold tumors.

Neuromodulation strategy	Mechanism of action	Immune-modulatory effects	Synergy with ICIs	Representative references
CP-AMPAR Antagonists (e.g., NASPM)	Blocks calcium influx via CP-AMPARs, disrupting glutamatergic signaling between neurons and tumor/glial cells.	Alleviates T cell exhaustion, inhibits recruitment of Tregs/MDSCs, restores antigen presentation.	Reprograms TME, enhances tumor immunogenicity, facilitates effective T cell activation.	(40, 72, 73)
Low-frequency rTMS (<1 Hz)	Non-invasive suppression of PI3K/AKT and ERK/JNK pathways; alters sympathetic output.	Promotes apoptosis, modulates norepinephrine-mediated immune suppression, may increase T cell infiltration.	Alters neuroimmune axis to sensitize tumors to ICIs.	(5, 77, 79)
Deep Brain Stimulation (DBS)	Targeted central excitability modulation; reduces IL-6 and caspase-3 levels.	Exhibits anti-inflammatory and anti-apoptotic effects; may relieve neuroinflammatory stress in CNS tumors.	Enhances immune tone, supports sustained antitumor immunity.	(79, 80, 82)
Combined Neuromodulation + ICI Therapy	Dual-targeting of neural and immune pathways.	Synergistic immune activation, reversal of immune exclusion, potential memory T cell formation.	Particularly beneficial for high PD-L1, low ICI-response tumors or CNS-involved cancers.	(54, 74, 75)
Biological Vectors (e.g., Ad-TD-nsIL12, DMAMCL)	Induce direct tumor cell death (e.g., oncolysis or mitochondrial damage); sustain IL-12 expression or modulate NF-κB–mediated inflammation.	Increase CTL infiltration, reduce immunosuppressive cells, reshape cold TIME to responsive state.	Transform immunologically “cold” gliomas into “hot” tumors, support long-term immune responses.	(83, 84)

### 5.2 Observations on the linkage between epilepsy control and immune response

Seizure activity is frequently accompanied by pronounced neuroinflammatory responses, characterized by microglial activation, peripheral T cell infiltration, and elevated levels of proinflammatory cytokines (85). Accumulating evidence has shown sustained increases in IL-1β, TNF-α, and IL-6 within brain parenchyma and cerebrospinal fluid of individuals with epilepsy, implicating these mediators not only in seizure initiation and propagation but also in the establishment of a self-reinforcing “seizure–immune” feedback loop via enhanced neuronal excitability (86, 87). This bidirectional interplay suggests that epilepsy should not be viewed solely as a disorder of aberrant neuronal discharges, but rather as a complex condition intricately linked to immune dysregulation. As such, therapeutic strategies targeting inflammatory and immune pathways—particularly those capable of modulating both neuronal and immune cell function—are gaining increasing attention. Valproic acid (VPA), a widely used broad-spectrum antiepileptic drug, has attracted interest for its immunomodulatory properties beyond seizure control. As a classical histone deacetylase (HDAC) inhibitor, VPA exerts multifaceted effects on immune signaling. It suppresses HDAC3 activity, thereby enhancing acetylation of transcription factors signal transducer and activator of transcription 1(STAT1) and NF-κB, promoting the phenotypic shift of microglia from proinflammatory M1 to anti-inflammatory M2 states (88). In parallel, VPA reduces the expression of key proinflammatory mediators such as TNF-α, IL-1β, and IL-6, alleviating neuroinflammation (88). Additionally, VPA upregulates immune metabolic genes including immune responsive gene 1(IRG1), increasing the production of its downstream metabolite itaconate and activating the nuclear factor erythroid 2–related factor 2(Nrf2) antioxidant pathway—an effect that contributes to maintaining redox and immune homeostasis at both central and systemic levels (89).

This dual function of VPA—as both an antiepileptic and immune-regulating agent—has been validated in a range of disease models. In hypertensive rats, long-term VPA administration attenuated cardiac oxidative stress and inflammation, independent of its effects on blood pressure (90). In cAMP response element modulator (CREM) transgenic mice, VPA delayed the onset of atrial fibrillation, reduced atrial remodeling, and reversed dysregulation of Ras homolog family member A (RhoA) and mitochondrial oxidative phosphorylation pathways (90). In experimental epilepsy, VPA has been shown to modulate CD4^+^/CD8^+^ T cell ratios, enhance Treg populations, and mitigate immune-mediated neuroinflammatory damage (91). These findings collectively support the therapeutic potential of VPA to not only stabilize neuronal excitability and suppress seizures, but also to reshape the immune microenvironment. Such a dual-action profile positions VPA as a promising candidate for treating inflammation-driven epilepsy and neuroimmune comorbidities.

While VPA exemplifies a systemically available dual-acting agent, other approaches such as CP-AMPAR inhibition offer localized neuromodulatory-immune benefits. However, the clinical application of CP-AMPAR antagonists must be approached with caution due to their involvement in normal synaptic transmission and cognitive processes. CP-AMPARs are enriched in hippocampal and cortical circuits where they contribute to excitatory synaptic plasticity and memory formation (92–95). Systemic blockade may therefore risk cognitive or behavioral side effects. To mitigate this, emerging strategies aim to achieve tumor-selective inhibition via nanoparticle delivery systems, or through local administration such as convection-enhanced delivery (CED), which minimizes off-target exposure. Additionally, low-dose or transient inhibition of CP-AMPARs has demonstrated immunomodulatory benefits in glioma models with limited neurological impairment, supporting a favorable therapeutic window (**Table 2**).

**Table 2:** Representative pathways of the neuron–immune–tumor axis in glioma and their potential therapeutic targets.

Tumor component	Signaling molecule	Role in tumor progression	Therapeutic target	References
Neuron → Glioma Cell	NLGN3	Activates PI3K–mTOR signaling, linked to glioma growth	Target NLGN3 or PI3K–mTOR axis	(10, 14, 32)
Neuron → Glioma Cell	BDNF → TrkB	Linked to AMPAR expression and invasiveness	Block BDNF–TrkB signaling	(11, 21, 53)
Neuron → Glioma Cell	Glutamate (via CP-AMPAR)	Linked to depolarization and immune evasion	CP-AMPAR antagonist NASPM	(11, 13, 73)
Neuron → Immune Cells	Norepinephrine	CCL2-mediated recruitment of Tregs/MDSCs	Modulate sympathetic activity or β-adrenergic receptors	(5, 33)
Neuron → TME Immune Cells	BDNF, IL-1β, PGE2	Shapes suppressive phenotype, promotes exclusion	Inhibit proinflammatory mediators or remodel the TME	(11, 47)
Glioma Cell → T Cells	PD-L1 upregulation (via AMPAR signaling)	Linked to T cell suppression and immune evasion	Combination of ICIs and AMPA blockade	(11, 37, 41)

## 6 Challenges and prospects

While this review emphasizes the predominantly immunosuppressive influence of neuronal activity in gliomas, it is worth noting that neuronal signals may exert bidirectional immunological effects depending on context. For example, certain studies have shown that sympathetic neural activity can enhance antigen presentation and dendritic cell priming under specific conditions, potentially promoting anti-tumor responses. Similarly, BDNF has been linked to microglial modulation and might support immune surveillance in non-malignant contexts (96, 97). These findings underscore the context- and tissue-specific nature of neural influence on the immune system, and highlight the importance of precise spatial and temporal modeling in future investigations. In this regard, animal models—particularly murine systems—have played an indispensable role in elucidating the mechanisms of neuro–immune interaction. Their well-defined genetic backgrounds and experimental accessibility make them ideal platforms to explore how neural signals modulate immune cell differentiation, migration, and functional programming. Reproducible insights have emerged from models simulating sympathetic activation, Notch-mediated contact signaling, and neuron–immune co-culture systems. However, translating these findings to human applications remains a major challenge. Interspecies differences in neural architecture, immune cell ontogeny, and microbiota exposure often limit the predictive value of murine data (98). Compounding this issue, most neuromodulatory strategies rely on systemic inhibition—such as whole-brain electrical stimulation or broad receptor blockade—which risks off-target effects including cognitive impairment and metabolic disturbance. These limitations underscore the need for precise, context-specific neuromodulation. Promising directions include tumor-selective neuronal targeting, programmable stimulation platforms, and neuron-specific delivery vectors such as ligand–receptor engineering or localized biomaterial systems.

Future strategies should also integrate electrophysiological and immune profiling. Combining neurophysiological modalities—such as multi-channel cortical recordings or calcium imaging—with single-cell immune omics (including T cell receptor (TCR)/B cell receptor (BCR) repertoire sequencing and spatial proteomics) may help decode how specific neural signals shape immune cell states within the TME. Conversely, understanding how immune perturbations modulate neuronal excitability could uncover reciprocal control axes. Finally, circuit-level dissection remains limited. Despite evidence that neuron-derived cues like NLGN3 or BDNF regulate immune landscapes, the anatomical wiring of tumor-infiltrating neural circuits remains elusive. Techniques such as viral tracing, connectomics, and *in vivo* optogenetics may help reconstruct this architecture.

**Figure 3:**
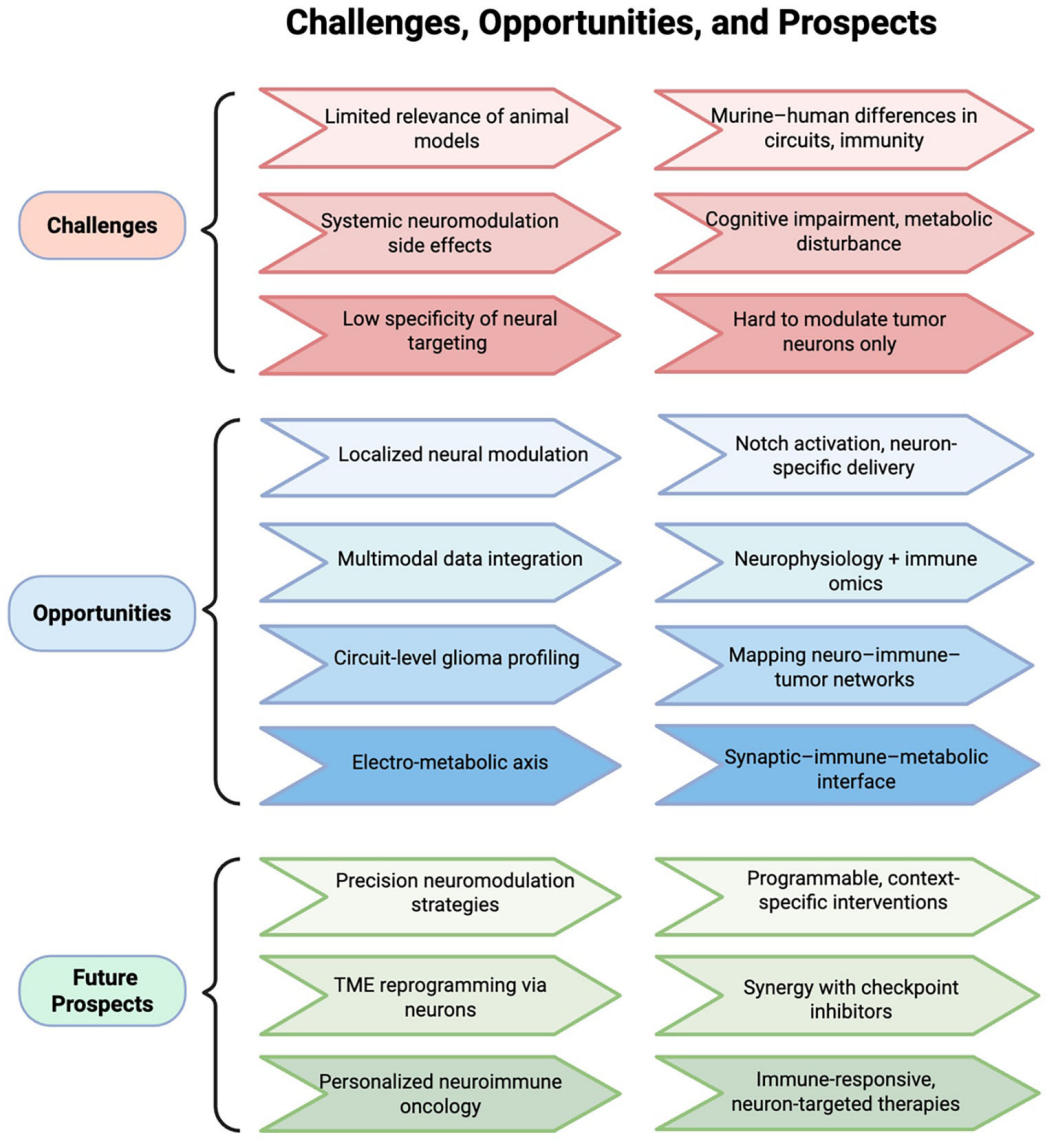
Challenges and prospects in neuro–immune–tumor modulation. The electro-metabolic axis refers to neuron-driven excitatory activity that reprograms metabolism and impairs immune surveillance in gliomas. The neuro–immune–tumor axis denotes the bidirectional regulatory loop linking neurons, immune cells, and tumor cells.

Altogether, overcoming these challenges will require human-relevant models, multimodal analysis frameworks, and cross-disciplinary innovation (**Figure 3**).

## 7 Conclusion

The neuron–immune–tumor axis is increasingly recognized as a critical regulatory pathway in glioma progression and immune evasion. Neuronal signals, including neurotransmitters and neurotrophic factors such as NLGN3, BDNF, glutamate, and norepinephrine, can alter immune cell phenotypes and contribute to the formation of an immunosuppressive tumor microenvironment. This review highlights the emerging role of neural activity in shaping antitumor immunity and summarizes current strategies targeting neuron-mediated pathways, including AMPA receptor inhibition, neuromodulation, biologically engineered immunotherapeutics, and the repurposing of antiepileptic agents. However, many aspects of spatiotemporal neural–immune interactions remain poorly understood. Future studies that integrate electrophysiological monitoring, organoid-based modeling and immune profiling may help elucidate context-dependent regulatory mechanisms and guide the development of precise, mechanism-based multimodal therapies to enhance immunotherapy efficacy in gliomas.

## Glossary

A2AR: adenosine A2A receptor
ACh: acetylcholine
α7 nAChRs: α7 nicotinic acetylcholine receptors
Ad-TD-nsIL12: oncolytic adenovirus with triple deletions expressing non-secreting interleukin-12
AHR: aryl hydrocarbon receptor
AKT: protein kinase B
AMPAR: α-amino-3-hydroxy-5-methyl-4-isoxazolepropionic acid receptor
AN: anterior nucleus
BCR: B cell receptor
BDNF: brain-derived neurotrophic factor
CAFs: cancer-associated fibroblasts
CaMKII: calcium/calmodulin-dependent protein kinase II
CCL2: C–C motif chemokine ligand 2
CD39: ectonucleoside triphosphate diphosphohydrolase-1
CD73: ecto-5’-nucleotidase
CED: convection-enhanced delivery
CP-AMPARs: calcium-permeable α-amino-3-hydroxy-5-methyl-4-isoxazolepropionic acid receptors
CREM: cAMP response element modulator
DBS: deep brain stimulation
EGF: epidermal growth factor
EPSCs: excitatory postsynaptic currents
ERK: extracellular signal-regulated kinase
GABA: gamma-aminobutyric acid
GBM: glioblastoma
HDAC: histone deacetylase
ICIs: immune checkpoint inhibitors
IDH: isocitrate dehydrogenase
IFN-γ: Interferon-gamma
IL-1β: interleukin-1 beta
IL-6: interleukin-6
IL-10: interleukin-10
IL-12: interleukin-12
iPSC: induced pluripotent stem cells
IRG1: immune responsive gene 1
JNK: c-Jun N-terminal kinase
MDSCs: myeloid-derived suppressor cells
MMPs: matrix metalloproteinases
NASPM: 1-naphthylacetyl spermine trihydrochloride
NF-κB: nuclear factor kappa-light-chain-enhancer of activated B cells
NLGN3: neuroligin-3
Nrf2: nuclear factor erythroid 2–related factor 2
PD-L1: programmed death-ligand 1
PGE2: prostaglandin E2
PI3K: phosphoinositide 3-kinase
mTOR: mechanistic target of rapamycin
rTMS: repetitive transcranial magnetic stimulation
RhoA: Ras homolog family member A
ROS: reactive oxygen species
STAT1: signal transducer and activator of transcription 1
STN: subthalamic nucleus
TAMs: tumor-associated macrophages
TAP1: transporter associated with antigen processing 1
TAP2: transporter associated with antigen processing 2
TCR: T cell receptor
TGF-β: transforming growth factor beta
TIME: tumor immune microenvironment
TME: tumor microenvironment
TNF-α: tumor necrosis factor alpha
TrkB: tropomyosin receptor kinase B
Tregs: regulatory T cells
VPA: valproic acid.
